# m6A RNA methylation in mammalian oocytes and preimplantation embryos: implications for developmental competence

**DOI:** 10.3389/fvets.2026.1883063

**Published:** 2026-08-27

**Authors:** Chiara Cosseddu, Sara Succu, Francesca Mossa, Adele Frau, Maria Grazia Palmerini, Sergio Ledda, Daniela Bebbere

**Affiliations:** 1 Obstetrics and Gynecology Clinics, Department of Veterinary Medicine, University of Sassari, Sassari, Italy; 2 Anatomy Section, Department of Veterinary Medicine, University of Sassari, Sassari, Italy; 3 Department of Life, Health and Environmental Sciences, University of L'Aquila, L'Aquila, Italy

**Keywords:** embryo, epitranscriptomics, gene regulation, m6A RNA methylation, oocyte developmental competence

## Abstract

Chemical modifications of RNA molecules, collectively referred to as epitranscriptomics, have emerged as a critical layer of post-transcriptional gene regulation. Among these modifications, N6-methyladenosine (m6A) plays a key role in modulating RNA stability, localization, and translational efficiency without altering the underlying nucleotide sequence. In recent years, epitranscriptomics has been increasingly recognized for its involvement in mammalian reproductive processes. In mammalian oocytes, which undergo an extended period of transcriptional quiescence and rely predominantly on post-transcriptional regulation of maternally stored transcripts, RNA modifications are essential for proper gene expression control. During oocyte maturation and early pre-implantation embryo development, epitranscriptomic mechanisms ensure the timely turnover and translational regulation of maternal mRNAs, thereby supporting the correct execution of the maternal-to-zygotic transition. The proper accomplishment of these processes is crucial for the acquisition of developmental competence. Reversible RNA modifications therefore add an additional layer of complexity to the regulation of gene expression in oocytes and early embryos. This mini-review summarizes current knowledge on the role of m6A RNA methylation in mammalian oocyte developmental competence and early embryogenesis, highlighting recent experimental evidence and discussing potential implications for fertility and assisted reproductive technologies.

## 1 Introduction

*In vitro* embryo production plays an increasingly important role in both medicine and agriculture, by improving assisted reproductive technology (ART) outcomes for treating human infertility and selecting superior traits in key agricultural species.

Determinants of embryo developmental competence are mainly rooted in the oocyte. Unravelling the biological pathways responsible for the acquisition of proper oocyte and embryo developmental competence may increase embryo production efficiency and facilitate the use of ARTs.

In metazoans, oocyte maturation, fertilization and early embryo development happen in the absence of *de novo* transcription (1). During this transcriptionally quiescent window, the molecules required to support development are stored as maternal mRNAs within the oocyte, and gene expression is mainly regulated at the post-transcriptional level (2, 3). Such maternal control lasts until activation of the embryonic genome (EGA), when mRNAs of maternal origin are progressively degraded and replaced by the molecules synthesized by the embryo (4). Maternal transcript turnover does not occur uniformly across all molecules: some transcripts undergo rapid degradation, whereas others remain stable until the morula stage. As a consequence, specific maternal proteins may be undetectable before oocyte maturation, appear in mature oocytes and persist during the early stages of embryonic development (4). In mammals, the timing of EGA is species-specific and occurs at different developmental stages; major EGA occurs at the 2-cell stage in mice (5), the 8–16 cell stage in ruminants (6) and the 4–8 cell stage in human embryos (7, 8).

Thus, the molecular endowment of the oocyte, the transcriptome, is critical to complete the complex mechanisms underlying oocyte to embryo transition. The transcriptional quiescence needs complex post-transcriptional and post-translational mechanisms to achieve a precise and timely recruitment of maternal RNAs for translation or degradation, in order to support proper meiotic maturation, fertilization, and reprogramming of the nascent genome (1).

Post-transcriptional regulation in oocytes and early embryos involves multiple mechanisms. During oocyte growth, maternal transcripts are either immediately used to support oocyte functions or stored for later use during the transcriptional silence (9). To this aim, mRNAs are post-transcriptionally modified to become dormant and be later recruited for translation. For instance, cytoplasmic polyadenylation regulates poly(A) tail length, influencing mRNA stability and translation (10). Additionally, specific sequences within transcript 3′ UTRs, along with RNA-binding proteins and microRNAs, further control transcript fate (11–13). Spatial compartmentalization within the oocyte cytoplasm also contributes to the control of gene regulation. Recently, the subcortical maternal complex (SCMC), a maternal-effect protein complex required for early embryo development (14, 15), and cytoplasmic lattices (CPLs), abundant filamentous structures in the oocyte cytoplasm (16, 17), have been redefined as components of a single supramolecular organization playing a role in regulation of maternal molecules. This SCMC/CPLs complex functions as a storage platform for maternal proteins in the oocyte, providing a spatial framework for their sequestration, protection and organization until they are required during the oocyte-to-embryo transition.

Over the past years, the epitranscriptome has emerged as an additional layer of post-transcriptional regulation of maternal transcripts. This term refers to the complete set of chemical modifications on RNA molecules within a cell, particularly mRNAs, that regulate RNA stability, translation, and degradation without altering the underlying nucleotide sequence (18, 19). Analogously to DNA, RNA is dynamically modified by the attachment of chemical groups, adding a previously unappreciated layer of regulatory control. To date, more than 150 distinct chemical modifications have been identified in various types of cellular RNAs, including protein-coding mRNAs as well as non-coding RNAs (20–22). In eukaryotic mRNAs, internal modifications, distinct from the canonical 5′ cap and 3′ poly(A) tail, include N6-methyladenosine (m6A), N1-methyladenosine (m1A), 5-methylcytosine (m5C), 5-hydroxymethylcytosine (5hmC), N6,2′-O-dimethyladenosine (m6Am), and 7-methylguanine (m7G) (23, 24). These modifications provide a versatile and dynamic mechanism for modulating RNA fate, adding complexity to post-transcriptional gene regulation during critical developmental windows such as oocyte maturation and early embryogenesis.

Among these, N6-methyladenosine (m6A) is the most abundant and conserved internal modification of mRNAs and long non-coding RNAs, exerting broad effects on post-transcriptional gene expression in eukaryotes (25). In mammals, m6A marks are non-randomly distributed and occur preferentially at the RRACH motif ([G/A/U][G > A]m6AC[U > A > C]), with enrichment in 3′ untranslated regions and near stop codons (26). Modulation of m6A levels regulates multiple aspects of mRNA metabolism, including mRNA stability, translation, pre-mRNA processing, and subcellular localization (27, 28).

In the oocyte and early embryo, m6A regulates stability or degradation of maternal RNAs and determines their fate, thereby affecting oocyte developmental competence (29, 30). m6A patterns are dynamically remodeled throughout oocyte-to-embryo transition and their effect is context-dependent. In some cases m6A may favor RNA stability or translation, whereas in other cases it promotes RNA decay and maternal mRNA clearance. In mouse, m6A-modified RNAs had a significantly increased expression from MII oocytes to early embryos, suggesting a protective role during the MII-to-zygote transition, but are subsequently degraded more rapidly than unmodified transcripts at the early and late 2-cell stages (31). These findings suggest that m6A coordinates maternal RNA fate in a developmental stage-dependent manner. Although m6A dynamics during oogenesis and early embryo development remain incompletely understood, these evidence supports a key role for this modification in coordinating maternal RNA fate, with important implications for oocyte competence and female fertility (32).

## 2 m6A methylation-related proteins: writers, readers and erasers

**Figure 1:**
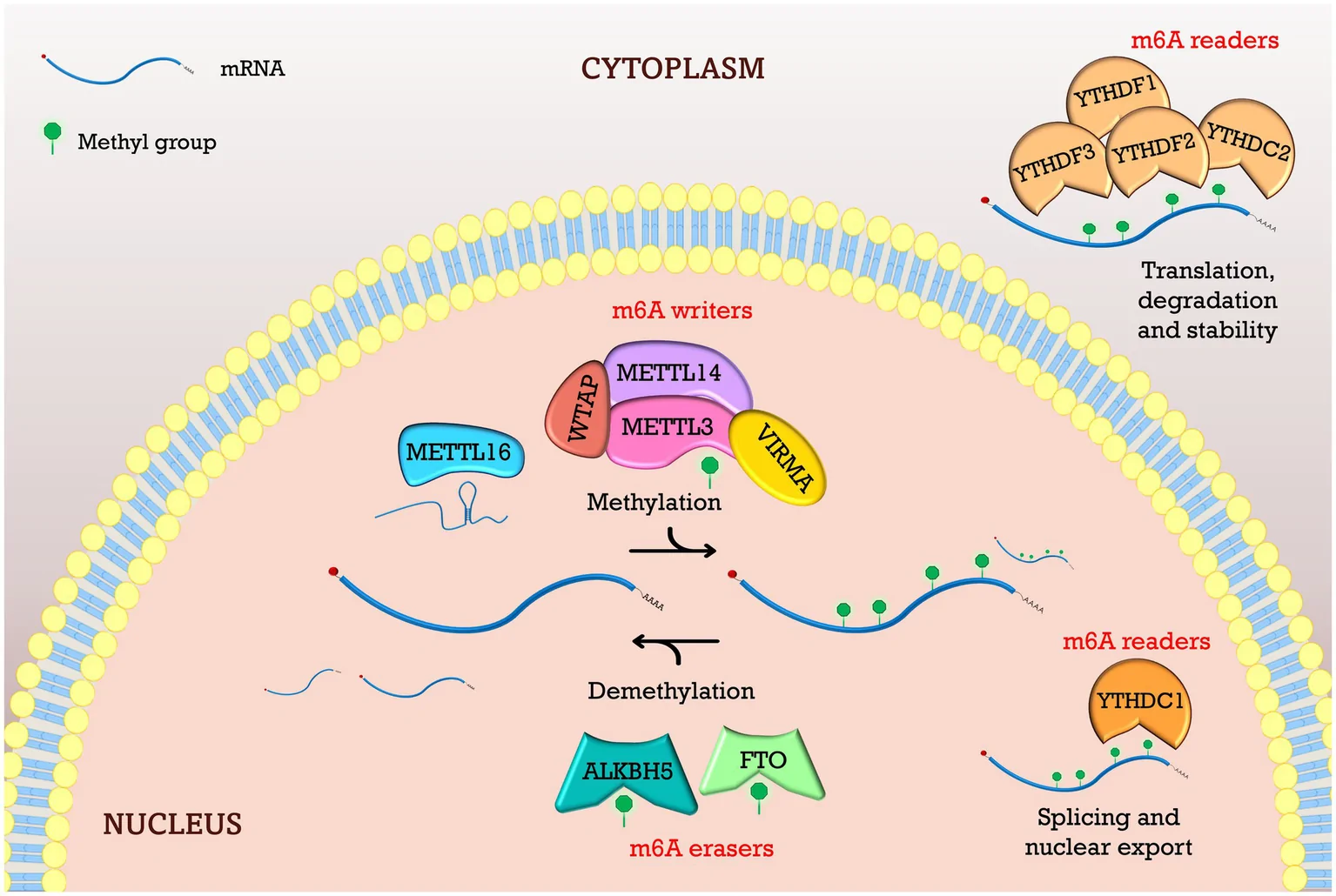
Schematic representation of reversible m6A RNA methylation and m6A-related proteins. In the nucleus, “writers” (METTL3, METTL14, WTAP, and VIRMA) mediate m6A deposition on transcripts, while METTL16 targets specific hairpin motifs. “Erasers” (ALKBH5 and FTO) remove m6A modifications from RNA. M6A “readers” include nuclear (YTHDC1) and cytoplasmic binding proteins (YTHDC2 and YTHDF1–3), which mediate the downstream fate of m6A-modified RNAs.

Similar to DNA methylation, RNA methylation acts as a dynamic, reversible regulator of gene expression. The deposition and removal of the methyl groups are mediated by three different classes of proteins, which are defined as writers (enzymes that add the methyl group to the N6 position of adenosine, e.g., METTL3 and METTL4), erasers (enzymes that remove the methyl group, e.g., FTO and ALKBH5), and readers (proteins that bind to m6A marks and activate downstream events, e.g., YTHDF and YTHDC families; Figure 1). Modulation of the abundance and activity of these proteins regulates m6A RNA methylation.

### 2.1 m6A writers

m6A is installed co-transcriptionally on nascent mRNAs by a multicomponent methyltransferase complex, commonly referred to as the “writer” complex. Its core is formed by methyltransferase-like 3 (METTL3), which provides the catalytic activity, and methyltransferase-like 14 (METTL14), which acts as a structural scaffold that stabilizes the complex and enhances RNA substrate recognition and binding (33, 34). Although both proteins contain methyltransferase domains, only METTL3 is catalytically active, as it binds the methyl donor S-adenosylmethionine (SAM), whereas METTL14 primarily facilitates substrate positioning and improves the efficiency and specificity of methylation (34, 35).

Beyond the METTL3–METTL14 heterodimer, the full writer complex includes several auxiliary components that are essential for proper localization and function. Among these, Wilms tumor 1- associating protein (WTAP) binds to METTL3/14 and is required for optimal substrate recruitment and localization of the complex (36); Vir like m6A methyltransferase associated (VIRMA, also known as KIAA1429) directs methylation preferentially to 3′UTRs and near stop codons, influencing mRNA stability and translational regulation (37). Functionally, this complex deposits m6A marks on pre-mRNAs within a conserved consensus sequence (RRACH motif), influencing multiple aspects of RNA metabolism (36). Mettl3-mediated m6A deposition plays distinct roles during oocyte maturation and early embryogenesis, promoting mRNA degradation in GV oocytes and regulating transcript stability during the transition from the MII stage to the zygote (31). Mettl14 and Virma are implicated in alternative splicing (38, 39). The co-transcriptional nature of m6A deposition allows tight coupling between transcription and post-transcriptional regulation, enabling rapid and fine-tuned control of RNA fate during key developmental processes.

METTL16 represents an additional m6A writer with distinct substrate specificity compared to the canonical METTL3/METTL14 complex. It preferentially methylates structured RNA elements, such as hairpin motifs, in specific targets including U6 small nuclear RNA, linking m6A deposition to the regulation of cellular S-adenosylmethionine (SAM) homeostasis (40). METTL16 has been detected in both nuclear and cytoplasmic compartments, where it contributes to multiple aspects of RNA metabolism (41), including post-transcriptional modification, RNA stability (42), and translational control (43). Functional studies have highlighted a critical role for METTL16 in early embryonic development. Genetic ablation of *METTL16* in mice results in severe developmental defects: although fertilized embryos can initially progress to the blastocyst stage, they exhibit impaired post-implantation development and fail to sustain normal growth after transfer into recipient females (44). These findings indicate that METTL16-mediated m6A regulation is essential for embryonic viability and proper developmental progression.

### 2.2 m6A readers

m6A-modified RNAs are recognized by reader proteins that decode m6A patterns and translate them in gene expression regulation. A prominent class contains the YT521-B homology (YTH) domain, including YTHDF1–3 and YTHDC1–2. Cytoplasmic YTHDF2 promotes the decay of target transcripts by recruiting the CCR4-NOT deadenylase complex (25). YTHDF1 enhances translation of methylated transcripts, whereas YTHDF3 coordinates both translation and decay with YTHDF1/2 (45).

In the nucleus, the reader YTHDC1 modulates pre-mRNA splicing by recruiting splicing factors, facilitates nuclear export, and accelerates transcript decay as required (27). Other readers, including IGF2BP proteins, stabilize m6A-marked transcripts, highlighting context-dependent functions of m6A readers (46).

### 2.3 m6A erasers

m6A erasers are enzymes that remove N6-methyladenosine (m6A) marks from RNA molecules, making this RNA modification reversible and dynamically regulated. By demethylating mRNAs, erasers allow fine-tuning of RNA stability, translation, and decay, providing a mechanism for rapid adjustment of gene expression in response to developmental cues or cellular signals (47).

The two best-characterized m6A demethylases are FTO (Fat mass and obesity-associated protein) and ALKBH5 (AlkB homolog 5). FTO was the first RNA demethylase identified and catalyzes the removal of the methyl group from m6A in mRNA both *in vitro* and in cells, thereby regulating RNA metabolism and translation (48). ALKBH5 specifically demethylates m6A in mRNAs, affecting RNA nuclear export, stability, and turnover, and plays crucial roles in germ cell development, including oocyte maturation and spermatogenesis (29, 49).

Other members of the AlkB family, such as ALKBH3, have been suggested to possess demethylase activity, although their physiological relevance in m6A regulation is less well established. Proper balance between m6A writers, readers, and erasers is essential during oogenesis and early embryogenesis, as dysregulation can impair maternal mRNA clearance and compromise developmental competence (3, 50) (Table 1).

**Table 1:** Effects of m6A-related protein alteration on embryo development in mammalian species.

Species	Gene	Alteration	Effects on embryo development
Mouse 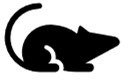	*Mettl3*	*Mettl3* CD	Failure in implantation and decidualization (76)
		*Mettl3* KD	No outgrowth and blastocyst formation; absence of m6A signal in the inner cell mass region (77)
	*Mettl14*	*Mettl14* KO	Embryonic or peri-natal death (78)
			Compromised post-implantation development and epiblast differentiation defects (39)
	*Mettl16*	*Mettl16* KO	Failure of embryo implantation (44)
	*Igf2bp2*	*Igf2bp2* KO	Early *in vitro* embryonic developmental arrest at the 2-cell-stage (79)
	*Ythdf2*	*Ythdf2* KO	Two cell-stage embryo arrest and cytokinesis defects (3)
	*Fto*	*Fto* KO	Reduced hatching efficiency; Impaired embryonic development with failure to reach E7.5 (80)
	*Alkbh5*	*Alkbh5* KO	Two cell-stage embryo arrest (29)
Human 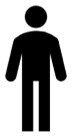	*YTHDC2*	*YTHDC2* BM	Early embryonic development arrest (81)
	*ALKBH5*	*ALKBH5* KD	Promotion of *in vitro* trophoblast invasion (82)
		*ALKBH5* OE	Inhibition of *in vitro* trophoblast invasion (82)
Pig 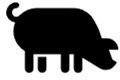	*METTL3*	*METTL3* KD	Reduction of total trophectoderm cell numbers and lineage formation (57)
		*METTL3* OE	Impaired blastocyst development and normal lineage allocation (57)
Goat 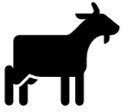	*YTHDF2*	*YTHDF2* KD	Decreased blastocyst rate (75)
	*METTL3*	*METTL3* KD	Decreased 8-cell and blastocyst rates in *in vitro* hypoxic condition (83)

KD: Knock-down; KO: Knock-out; OE: Overexpression; CD: Conditional deletion; BM: Biallelic Mutation.

## 3 m6A RNA methylation in oogenesis and oocyte developmental competence

Due to the transcriptional silence that occurs during oocyte maturation and early embryo development, post-trancriptional regulation in oocytes and embryos is highly complex and characterized by cell-specific mechanisms. In this context, the implication of m6A RNA in controlling maternal transcript utilization (leading to translation or degradation) was postulated and confirmed by several studies. Accumulating evidence indeed indicates that m6A represents a critical layer of post-transcriptional control governing maternal mRNA fate during oocyte growth (51), meiotic maturation (50), and the oocyte-to-embryo transition (52). m6A RNA methylation has been consistently detected in mammalian oocytes and preimplantation embryos, where it exhibits dynamic and stage-specific regulation (32). Its pattern was seen to be altered under non-physiological conditions such as parthenogenesis (53).

m6A RNA methylation has been implicated in several reproductive processes across species and reproductive tissues. For example, m6A levels in human granulosa cells vary according to patient age (54). Similarly, in sheep, m6A levels in cumulus cells have been associated with differences in oocyte developmental competence (55). Notably, m6A methylation-related proteins have been shown to support oocyte maturation and blastocyst development in both mice (56), goat (83), and pigs (57).

High-resolution epitranscriptomic studies have revealed the dynamic landscape of m6A during oocyte maturation and early embryo development, with stage-specific redistribution of methylation marks and selective targeting of transcripts involved in meiosis, RNA metabolism, and early developmental pathways (21, 31). These analyses showed that m6A modifications undergo extensive remodeling during key developmental transitions, influencing RNA stability, translation, and degradation in both oocytes and preimplantation embryos, and that methylated transcripts often exhibit differential translational and stability profiles compared with unmethylated counterparts.

In accordance, a recent study revealed the dynamic landscape of m6A methylome across human and mouse oocytes and early embryos, and showed that transcripts marked by m6A are generally expressed and translated at higher levels than unmarked transcripts, in both species (52). The Authors further reported that m6A marks clearly decrease in parallel with maternal RNA decay, but are gradually re-established through *de novo* methylation during EGA in both human and mouse early embryos (52).

Consistent with these findings, m6A methylation was shown to regulate the timely remodeling of the transcriptome by stabilizing a subset of maternal transcripts destined for Z-decay, thereby preventing their premature degradation, while promoting the timely clearance of 2C-specific transcripts and controlling the accumulation and turnover of transposable element-derived RNAs in mice (58). Maternal knockout of *Virma*, a component of the RNA m6A methyltransferase complex, in mouse oocytes resulted in failure of germinal vesicle (GV) breakdown and meiotic resumption (38), together with a reduced number of m6A-modified transcripts in GV oocytes and widespread transcriptome disorders. In line with this, VIRMA was demonstrated to be required for m6A deposition on maternal mRNAs that undergo decay after zygotic genome activation (58). Overall m6A abundance continuously decreased during the MZT, in parallel to transcript degradation.

In sheep, investigations of m6A RNA methylation dynamics during *in vitro* maturation (IVM) of cumulus–oocyte complexes (COCs), within a differential model of developmental competence, have revealed significant stage- and competence-associated changes in both oocytes and surrounding cumulus cells (55). Quantitative analyses showed that m6A levels, together with the transcript abundance of key m6A regulatory proteins, differ between COCs derived from adult and prepubertal donors, with these dysregulations becoming particularly evident after IVM (55). Notably, alterations in m6A are accompanied by coordinated changes in the expression of writers (e.g., METTL3, METTL14, VIRMA), readers (e.g., YTHDC1, YTHDF3), and erasers (e.g., ALKBH5, FTO) in both cellular compartments, and by alterations in m6A levels in cumulus cells. Overall, these findings suggest a functional link between m6A patterns and oocyte quality: altered expression of m6A regulatory proteins may affect m6A mediated regulation, potentially contributing to the reduced developmental competence observed in oocytes from prepubertal animals. However, it should be kept in mind that changes in the expression of m6A regulators do not necessarily imply transcript-specific changes in m6A deposition, nor do they demonstrate causal effects on oocyte quality or embryo developmental competence. Therefore further functional studies are required to confirm this hypothesis.

In this regard, functional studies in mice, based on the manipulation of the expression of m6A methylation-related proteins, have provided relevant insights into the role of m6A during oogenesis. Oocyte-specific ablation of *METTL3* in mice resulted in a global reduction of m6A-modified maternal mRNAs, potentially leading to defective folliculogenesis, accumulation of DNA damage, and failure to complete meiosis, ultimately resulting in infertility (59). Consistently, depletion of *METTL3* in fully grown oocytes impaired first polar body extrusion, disrupted spindle organization, and reduced global translational efficiency, ultimately compromising oocyte maturation (60). These phenotypes are consistent with an essential role of m6A in oogenesis and meiotic progression, suggesting that is required for proper developmental competence.

Beyond METTL3, additional components of the m6A machinery are essential for correct m6A establishment in oocytes. METTL14 and WTAP contribute to the proper deposition of m6A marks (33, 36), and are essential for proper mouse post implantation development (39, 61), while YTH domain-containing proteins mediate downstream functional outcomes (3). In particular, the cytoplasmic reader YTHDF2 has emerged as a key regulator of maternal mRNA clearance, promoting selective degradation of methylated transcripts and ensuring timely transcriptome remodeling during mouse oocyte maturation and early embryogenesis (3). This function is essential for the transition from a stable maternal transcript pool to a dynamically regulated transcriptome competent for EGA. In parallel, nuclear m6A readers such as YTHDC1, which regulate alternative splicing and RNA processing during oocyte development, have been implicated in the control of developmental competence. In mice, YTHDC1 is essential for oocyte growth and maturation, as Ythdc1-deficient oocytes are arrested at the primary follicle stage. Loss of YTHDC1 leads to widespread alterations in RNA processing, including extensive changes in alternative polyadenylation that affect 3′ UTR length, as well as global defects in alternative splicing, ultimately compromising oocyte developmental potential (56).

The reversible nature of m6A modification further contributes to the fine-tuning of maternal RNA fate. The demethylase ALKBH5 has been shown to regulate RNA stability and to be required for maintaining oocyte quality, with altered ALKBH5 activity leading to defects in RNA metabolism and reduced developmental competence (29).

Together, these findings support a model in which the dynamic interplay between m6A methylation and demethylation regulates transcript dosage, coordinating mRNA storage, translation, and degradation in transcriptionally silent oocytes. Beyond stabilizing transcripts, m6A enhances the translation efficiency of specific maternal mRNAs, a role conserved across mammalian species and early developmental stages (21, 62). By modulating the selective recruitment of stored maternal transcripts, m6A directly shapes oocyte developmental competence. Its regulation of maternal mRNA degradation during meiotic maturation and after fertilization ensures the proper timing of the maternal-to-zygotic transition, when embryonic transcription begins and maternal transcripts are cleared.

## 4 m6A RNA methylation in mammalian preimplantation embryos

Following fertilization, mammalian embryos undergo a highly coordinated developmental program characterized by transcriptional quiescence, progressive maternal mRNA clearance and EGA. m6A RNA methylation has emerged as a key regulator of transcriptome remodeling also within this context, during preimplantation development, ensuring the temporal and quantitative control of gene expression required for successful progression to the blastocyst stage.

Transcriptome-wide analyses have demonstrated that m6A is widely distributed across all stages of preimplantation development, from the zygote to the blastocyst, with thousands of methylated transcripts identified at each stage and a conserved enrichment near stop codons and within 3′UTRs (63, 64). Notably, m6A landscapes are highly dynamic, with substantial remodeling occurring during the transition from the zygote to the two-cell stage, coinciding with the onset of major EGA in mouse. This stage is characterized by both gain and loss of m6A marks on distinct subsets of transcripts, reflecting coordinated processes of maternal RNA degradation and zygotic gene activation (63).

Functionally, m6A plays a central role in orchestrating the MZT, a critical developmental window during which control shifts from maternally inherited transcripts to the embryonic genome. m6A modification of maternal mRNAs has been shown to promote their timely degradation while simultaneously stabilizing and enhancing the translation of a subset of transcripts required after fertilization (62). This dual role highlights the context-dependent function of m6A in balancing RNA decay and translation during early embryogenesis.

The activity of m6A reader proteins is particularly crucial during this developmental timeframe. The cytoplasmic reader YTHDF2 mediates the clearance of methylated maternal transcripts and is maternally required for early embryonic development; its deficiency results in impaired transcriptome remodeling and developmental arrest at early cleavage stages (3). More recently, additional YTHDF family members have been shown to cooperatively regulate early embryonic development, including the control of retrotransposon expression, thereby safeguarding genome stability during cleavage stages (30). In parallel, nuclear readers such as YTHDC1 contribute to m6A-mediated regulation of RNA metabolism during MZT, further supporting the coordinated control of gene expression at both nuclear and cytoplasmic levels (62).

Beyond maternal RNA clearance, m6A has also been implicated in the regulation of embryonic genome activation and early lineage specification in mouse (63). In particular, m6A-marked transcripts are enriched among genes involved in transcriptional regulation, cell proliferation, and developmental pathways activated during early embryogenesis, suggesting a role in promoting the establishment of totipotency and subsequent cell fate decisions (63). These m6A-modified transcripts also show increased targeting by microRNAs, indicating a coordinated interaction between epitranscriptomic and post-transcriptional regulatory networks in shaping the embryonic transcriptome.

Similarly, in early human embryos. Profiling of m6A marks suggests that methylated transcripts that are maintained across developmental transitions exhibit higher translation efficiency compared with unmodified RNAs, indicating that m6A can promote translation in conjunction with other regulatory mechanisms (21).

These findings highlight m6A RNA methylation as a central regulator of transcriptome dynamics across mammalian species, orchestrating RNA stability, translation, and decay to ensure proper developmental progression. As in oocytes, where m6A regulates maternal transcripts, it also plays a crucial role in preimplantation embryos, where maternal and embryonic mRNAs coexist. In both contexts, disruption of m6A pathways leads to defects in the MZT, impaired embryonic genome activation, and early developmental arrest, underscoring that m6A-mediated post-transcriptional control from oocyte to embryo is essential for establishing developmental competence.

## 5 m6A RNA methylation in the context of assisted reproductive technologies

Assisted reproductive technologies (ART) encompass a suite of procedures, including controlled ovarian stimulation, *in vitro* maturation (IVM), *in vitro* fertilization (IVF), embryo culture, cryopreservation, intracytoplasmic sperm injection (ICSI), and preimplantation genetic testing (PGT) that have made considerable advances in addressing infertility. While ongoing innovations continue to improve ART effectiveness, success rates remain limited by various factors, including oocyte quality, maternal age, genetic stability, underlying causes of infertility and overall reproductive health conditions.

Consequently, understanding the mechanisms underlying germ cell and early embryonic development is crucial for enhancing reproductive health and improving ART outcomes. Notably, ART procedures expose gametes and embryos to non-physiological environments. Such exposure, particularly during *in vitro* manipulation, was seen to perturb gene regulation in gametes and early embryos (65). Emerging evidence further suggests that epitranscriptomic regulation, especially m6A RNA modification, may also be affected, with potential consequences for oocyte competence and early embryonic development (21, 63). A deeper understand of the involved mechanisms and molecules is therefore essential to enhance reproductive outcomes. Although this field remains under active investigation and data in oocytes and embryos are still limited, recent studies indicate that ART-related stressors can influence the expression and activity of key epitranscriptomic regulators (writers, erasers, and readers) independently of global transcriptional changes, suggesting a direct impact on RNA methylation dynamics (66).

In porcine embryos produced by somatic cell nuclear transfer, *METTL3* expression and m6A methylation exhibited dynamic changes and differed from parthenogenetically activated embryos, highlighting the sensitivity of epitranscriptomic regulation to the mode of embryo production (67).

Further evidence supporting the susceptibility of the epitranscriptome to ART-associated interventions comes from studies on cryopreservation. In boars, cryopreservation has been shown to dysregulate the expression of m6A writers (METTL3 and METTL14), erasers (ALKBH5 and FTO), and readers (YTHDF2), leading to altered mRNA methylation patterns (68). These changes were associated with enrichment of highly methylated transcripts in metabolic pathways (e.g., ATP5F1, LDHA) and correlated with reduced sperm motility and increased apoptosis. Similarly, vitrification has been reported to exert long-term effects on mouse embryos, including reduced m6A levels in blastocysts, both in the inner cell mass and trophectoderm, alongside downregulation of *Mettl3* and *Mettl14* and upregulation of *Ythdc1* transcripts (69).

Altered abundance of m6A-related proteins can lead to changes in m6A deposition, which may in turn impact critical developmental processes, including maternal mRNA clearance, the timing of zygotic genome activation, and the coordinated regulation of early embryonic programs. These processes are essential for successful progression beyond the preimplantation stages and are tightly regulated under physiological conditions. High-resolution epitranscriptomic studies have shown that m6A marks are highly dynamic and stage-specific during normal development (21, 31), suggesting that disruptions to these patterns during *in vitro* procedures could impair developmental competence.

Collectively, these observations highlight the remarkable sensitivity of the epitranscriptome to ART procedures and suggest that RNA methylation marks, particularly m6A, may act as predictive biomarkers of oocyte and embryo quality, but also as potential targets for optimizing ART protocols. A deeper understanding of how ART influences m6A dynamics will be critical for refining culture conditions, hormonal stimulation strategies, and cryopreservation techniques, ultimately improving both short-term embryo viability and long-term reproductive outcomes.

## 6 Current gaps and future perspectives

Despite the growing body of evidence supporting a central role for m6A in oocyte biology, several key questions remain unresolved. First, the precise identification of stage-specific m6A-targeted transcripts during oocyte growth and meiotic maturation is still incomplete, largely due to technical limitations associated with the low RNA input obtained from oocytes. Although modern m6A-seq approaches are available, such methods generally rely on immunoprecipitation of fragmented RNA using affinity purified anti-m6A antibody and massively parallel sequencing. As a result, they allow the identification of transcript-wide m6A enrichment but do not provide single-nucleotide resolution or precise localization of individual modification sites (70).

The combination of MeRIP and RNA seq was applied to bovine oocytes during *in vitro* maturation, revealing m6A dynamic changes associated with changes in gene expression. However, the authors did not validate individual m6A targets identified by sequencing, due to the limited input material and the substantial RNA requirements for library preparation, corresponding to approximately 900 oocytes per stage (71). This highlights the technical challenges associated with epitranscriptomic analyses in low-input systems such as mammalian oocytes. The authors also noted that changes in m6A peak counts should be interpreted cautiously, as oocyte maturation occurs under transcriptional silencing and is accompanied by extensive remodeling of the maternal transcriptome, which may influence transcript abundance and peak detection. Thus, how these changes correlate with altered oocyte developmental competence remain to be elucidated (71). These findings were complemented by immunofluorescence staining, which revealed an increase in global m6A levels at the MII stage. This observation differs from the MeRIP-seq results because MeRIP-seq profiles m6A modifications specifically on poly(A) + mRNAs, whereas immunofluorescence detects m6A across the total RNA population within oocytes (71).

Single cell picoMeRIP-seq quantification was developed and benchmarked using picogram-scale input from oocytes and embryos by Li et al. (72). This approach showed that m6A is enriched around stop codons and within 3’UTR, reaching its highest levels in GV oocytes before decreasing in MII oocytes, zygotes and two-cell embryos, and then increasing again at the eight-cell and blastocyst (72). Nevertheless, this approach does not establish whether these dynamic changes in m6A are functionally linked to maternal mRNA degradation and/or other molecular events underlying the maternal-to-zygotic transition. As a consequence, the direct link between m6A deposition and the regulation of specific maternal mRNAs controlling developmental competence remains only partially defined (30, 55). Second, although the role of major regulators such as METTL3 and YTHDF2 has been functionally characterized, the contribution of other components of the m6A machinery, including additional readers and accessory proteins of the writer complex, is still under investigation, particularly in the context of mammalian oogenesis (62, 73). Moreover, the interplay between m6A and other post-transcriptional regulatory mechanisms, such as cytoplasmic polyadenylation, RNA-binding proteins, and microRNAs, remains largely unexplored, despite evidence that such mechanisms cooperatively influence maternal RNA metabolism during early development.

Another important gap concerns the integration of m6A dynamics with cellular compartmentalization within the oocyte, including RNA localization and storage in ribonucleoprotein granules, which are known to play a crucial role in translational control. In addition, oocyte-specific structures such as the Subcortical Maternal Complex, which contributes to cytoplasmic organization in oocytes and early embryos, may also participate in coordinating these processes, although their potential interplay with m6A-dependent regulation remains largely unexplored.

Furthermore, the extent to which m6A-mediated regulation differs between *in vivo* and *in vitro* conditions is still unclear, despite increasing evidence that assisted reproductive technologies, including IVM, may perturb the epitranscriptomic landscape (66). Finally, while emerging studies suggest that m6A alterations are associated with reduced oocyte quality and developmental competence, a causal relationship has not yet been fully established, particularly in large animal models and humans. The METTL3 protein shows high sequence homology across multiple species, including goats, sheep, cattle and humans, and lower homology to mice (74). Studies in large animal models may therefore provide a valuable bridge between murine studies and human biology. As mentioned above, reducing m6A levels using a competitive inhibitor impaired polar body extrusion and cumulus expansion in bovine oocytes (71). However, this m6A-reducing agent may also interfere with other methylation pathways, making it difficult to attribute the observed effects exclusively to m6A depletion (71). In goat, loss of METTL3 or of the m6A reader YTHDF2 impaired embryo development by altering maternal mRNA clearance and EGA (74, 75). However, METTL3 also altered the expression of key downstream targets involved in other epigenetic modifications essential for embryo development (74), suggesting that the observed effects cannot be attributed solely to the loss of m6A but may reflect broader epigenetic interactions. Addressing these gaps will be essential to determine whether modulation of m6A pathways could represent a viable strategy to improve oocyte competence and embryo development in assisted reproduction technologies.
